# His‐bundle pacing as an alternative to CRT in a patient with left bundle branch block, left ventricular dysfunction, and TAVI‐induced complete AV block

**DOI:** 10.1002/ccr3.4000

**Published:** 2021-03-06

**Authors:** Pedro Silva Cunha, João Pais, Mário Martins Oliveira

**Affiliations:** ^1^ Arrhythmology, Pacing and Electrophysiology Unit Cardiology Service, Santa Marta Hospital Central Lisbon Hospital University Center Lisbon Portugal

**Keywords:** cardiac resynchronization therapy, his‐bundle pacing, left bundle branch block, TAVI

## Abstract

Our clinical case supports the effectiveness of distal His‐Bundle pacing in obtaining ventricular resynchronization in patients with LBBB and left ventricular dysfunction, particularly in the context of post‐TAVI conduction disturbances.

## Graphical abstract

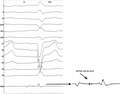

## 1 INTRODUCTION

Transcatheter aortic valve implantation (TAVI) has experienced an impressive growth in recent years. New‐onset conduction disturbances with need for permanent pacing are relatively common after TAVI. We report a case of a permanent pacemaker implantation in a patient with (previous) left bundle branch block (LBBB), left ventricular ejection fraction (LVEF) of 32%, and high degree atrioventricular block (AVB) induced after TAVI, with His‐bundle capture resulting in the correction of the LBBB and LVEF improvement in the early postoperative period.

Surgical aortic valve replacement has been the gold standard treatment for severe aortic stenosis. With aging and increasingly multimorbidity of the western world population, alternative and less invasive procedures were developed. Transcatheter aortic valve implantation (TAVI) was first introduced in 2002^1^ to further reduce surgical trauma and to avoid cardiopulmonary bypass.^2^ After this first event, an impressive increase in annual worldwide implantations was observed, due to a favorable ratio of risk to benefit.^3^ New‐onset conduction disturbances are relatively common after TAVI, with various reports in the literature documenting it in as much as 34.8% of patients at hospital discharge,^2^, ^3^ and with left bundle branch block (LBBB) being the most common significant conduction abnormality after TAVI (10.5%). His‐bundle pacing (HBP) is an effective alternative to right ventricular (RV) and biventricular pacing and is performed with the aim of maintaining a physiological pattern of ventricular activation, via the native His‐Purkinje system.^4^

## 2 CASE PRESENTATION

**FIGURE 1:**
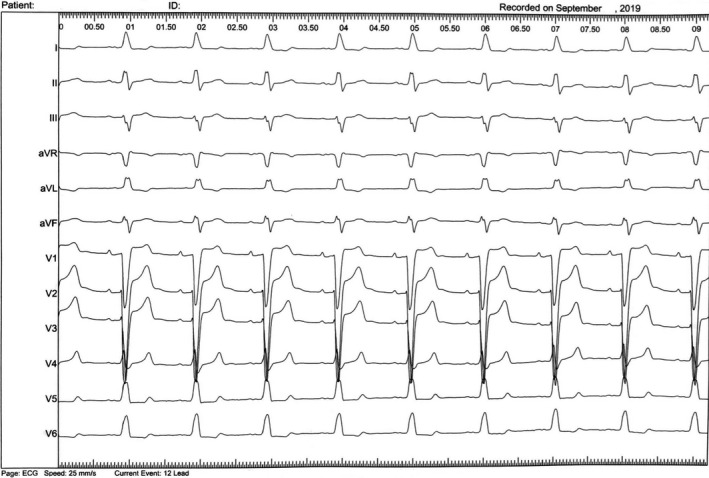
Baseline ECG tracing, showing complete left bundle brunch block (QRS width 150 mseg)

A 83‐year‐old man was electively admitted for intervention and management of severe aortic stenosis with New York Heart Association class III heart failure. The preoperative electrocardiogram (ECG) showed sinus rhythm with LBBB (wQRS 150 ms) (Figure 1). Transthoracic echocardiography documented a calcified aortic valve, with reduced cusp excursion, with valve area of 0.7 cm^2^ and mean gradient of 41 mm Hg. Left ventricular ejection fraction (LVEF) was 32%, with akinesia of the inferior wall and septum, and moderate mitral regurgitation.

**FIGURE 2:**
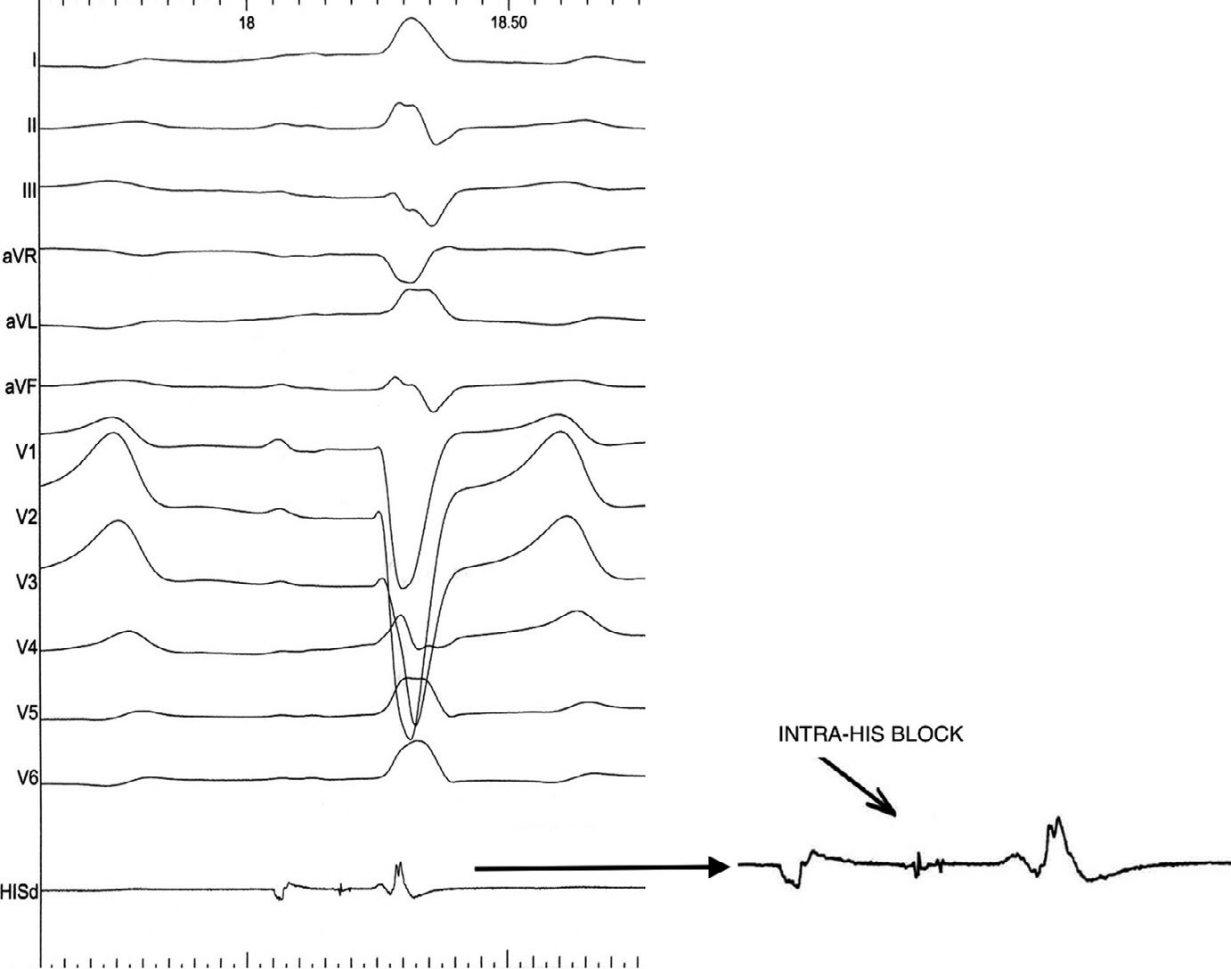
Intracardiac recording (with the pacing lead), depicting the intrahisian conduction delay (splitting of the His potential)

**FIGURE 3:**
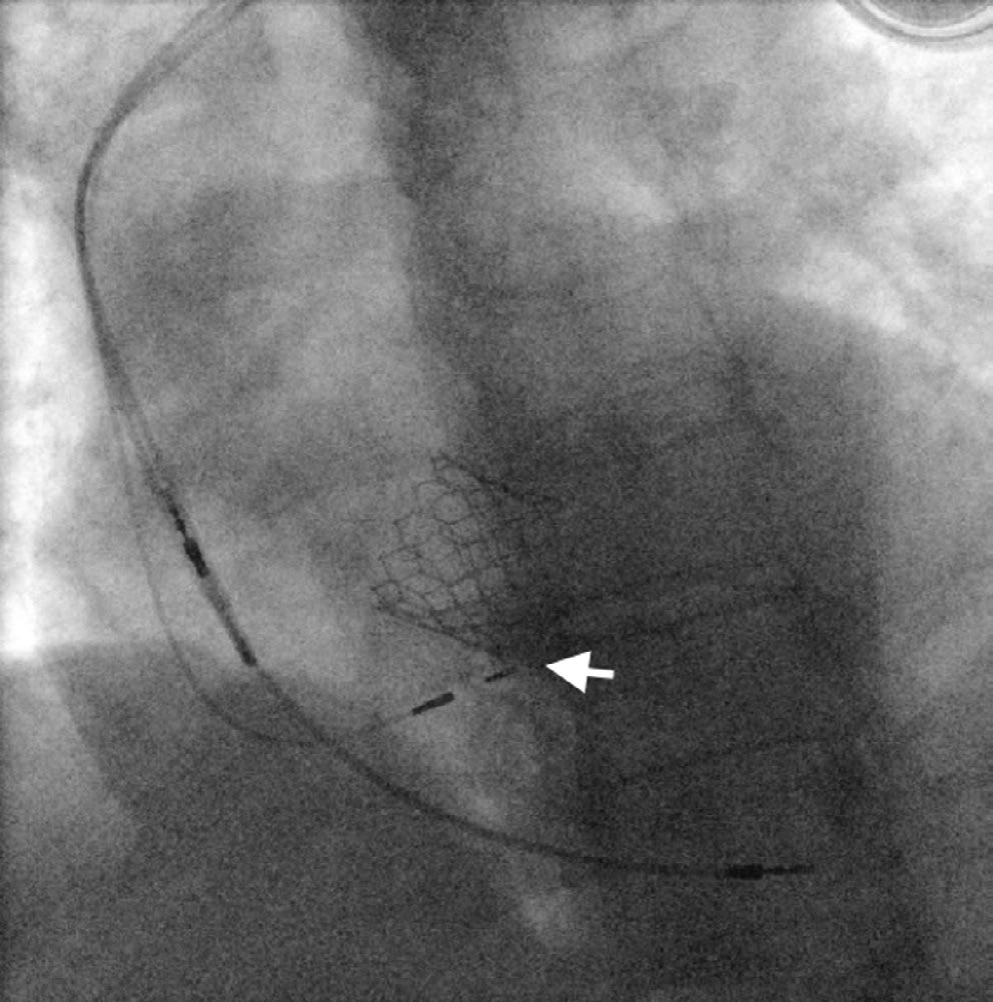
Fluoroscopy image showing the final position of the pacing leads. His lead (white arrow)

Using a left femoral approach, an Edwards SAPIEN 3 26mm Transcatheter Heart Valve was deployed without complications and with need of postdilatation. As per protocol, a temporary pacing wire was left at the RV apex. At the time of TAVI, the patient maintained LBBB. Within the first 24 hours post‐TAVI, the patient developed episodes of Mobitz 2 AVB and high‐grade AVB. In this clinical context, we decided to implant a permanent pacemaker (pacing without ICD due to advanced age and significant comorbidities). Via the left subclavian vein, 3 catheters were introduced (atrial lead, His capture lead, and RV apical lead as backup). For His pacing, a fixed curve C315 His sheath (Medtronic^®^) and a 3830 Select Secure lead (Medtronic^®^) was used. Operating the pacing lead alone, with unipolar mapping connected to the pacing system analyzer atrial channel (at a gain setting of 0.05 mV/mm and a sweep speed of 50 mm/sec), the intracavitary electrograms showed a slightly prolonged HV interval (62 ms), with a double His potential (Figure 2). Pacing with capture in the proximal His location was obtained at a threshold less than 1 V, but without recruitment of the left bundle branch. Correction of LBBB (Figure 3) occurred at 3 V at 1  ms. A Medtronic^®^ 4076 active fixation lead was then placed in apical RV, with a threshold 0.75 V at 0.5 ms, and a Medtronic^®^ 4076 lead was placed at the right atrial appendage, with a threshold 1.5 V at 0.5 ms.

The leads were connected to a Medtronic^®^ Serena biventricular generator (CRT‐P). RV lead was connected to the RV port for ventricular sensing and backup pacing (in case of loss of His lead capture), and His lead was connected to the left ventricular port. The device was programmed to DDD(R) biventricular pacing, with HBP (programed at 6 mV/1 ms) delivered before RV pacing (interventricular delay set to the maximum value of 80 ms).

**FIGURE 4:**
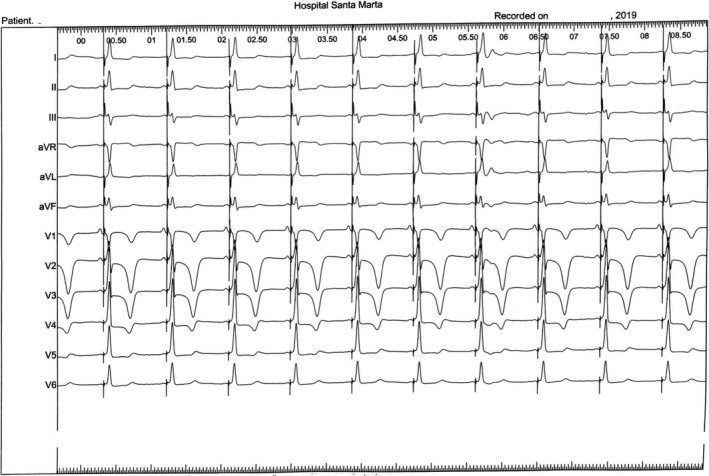
ECG tracing showing ventricular pacing with His‐bundle capture (nonselective) and QRS width of 90 ms

Paced QRS duration was 90 ms on the ECG (Figure 4), consistent with “His resynchronization”^5^ After the pacing spike, an initial pseudodelta wave is observed, due to the simultaneous capture of the neighboring myocardium and recruitment of the His‐Purkinje system.

Transthoracic echocardiography, repeated 48 hours after pacing implantation, showed improvement in overall systolic function (LVEF 44%), without evidence of desynchrony and a normal prosthetic valve function.

At 6 months follow‐up, the pacing threshold stabilized at 2 V at 1 ms

## 3 DISCUSSION

Transcatheter aortic valve implantation has revolutionized the management of aortic stenosis and become the standard of care for patients with aortic stenosis at prohibitive surgical risk, and the preferred treatment for many intermediate and high‐risk elderly patients.^6^

His‐bundle pacing has the potential to offer ventricular resynchronization, since significant reductions in QRS duration have been observed when stimulating the His‐Purkinje system in patients with LBBB.^7^

In the present case, the proximal His recording revealed a split His potential, which is highly suggestive of the presence of intrahisian disease. Pacing slightly distally from this site resulted also in correction of the LBBB (with an evident narrowing of the QRS width from 150 ms to 90 ms). In patients with LBBB, conduction system pacing can deliver cardiac resynchronization therapy by correcting BBB (multi‐layer block with bundle recruitment) to synchronize ventricular activation.^8^ “Bundle recruitment” refers to capture of the previously nonfunctional conduction fibers.

Recently,^9^ some authors proposed LBB direct pacing (with a lead traversing the interventricular septum), as an option with a lower pacing threshold, that also has the advantage of avoiding later deterioration at the proximal His‐bundle or AV node caused by the progression of AV conduction delay.

Our clinical case supports the potential effectiveness of HBP in obtaining ventricular resynchronization for patients with LBBB and left ventricular dysfunction, particularly in the context of post‐TAVI conduction disturbances.

## 4 CONCLUSION

Conduction abnormalities after TAVI are relatively common and frequently have indication for implantation of a permanent pacemaker. Although it may not be generalized to all post‐TAVI cases as the block induced mechanically by the valve may be more distal regarding the His‐Purkinge system, His pacing may be useful in the treatment of patients with LBBB and AV conduction disturbance after TAVI with the recruitment of the intrinsic conduction tissue and normalization of intraventricular conduction. This pacing modality reduces the electromechanical desynchrony associated with conventional right ventricular pacing, which could be potentially beneficial in the particularly vulnerable group of aortic stenosis patients treated with TAVI.

## CONFLICT OF INTEREST

The authors have no conflict of interest to declare.

## AUTHOR CONTRIBUTIONS

PSC and MMO: designed and directed the project. JP and PSC: collected the data. PSC: wrote the manuscript with support from JP and MMO.

## ETHICAL APPROVAL

Written informed consent was obtained from the patient for the publication of this case report.

## Data Availability

Patient data are available.
